# Healthcare resource utilization in patients on lipid-lowering therapies outside Western Europe and North America: findings of the cross-sectional observational International ChoLesterol management Practice Study (ICLPS)

**DOI:** 10.1186/s12944-020-01235-5

**Published:** 2020-04-07

**Authors:** Lieven Annemans, Joseph Azuri, Khalid Al-Rasadi, Ibrahim Al-Zakwani, Veronique Daclin, Florence Mercier, Nicolas Danchin

**Affiliations:** 1 Department of Public Health, Ghent University, Corneel Heymanslaan 10, 9000 Ghent, Belgium; 2 Maccabi Healthcare Services and Sackler Faculty of Medicine, Tel Aviv University, Tel Aviv, Israel; 3 College of Medicine & Health Sciences, Sultan Qaboos University, Muscat, Oman; 4 Sanofi-Aventis, Paris, France; 5 Stat Process, Paris, France; 6 Department of Cardiology, European Hospital Georges-Pompidou, Paris, France

**Keywords:** Dyslipidemia, Health resource, Disease management, Statins, Observational study

## Abstract

**Background:**

Few recent large-scale studies have examined healthcare consumption associated with dyslipidemia in countries outside Western Europe and North America.

**Methods:**

This analysis, from a cross-sectional observational study conducted in 18 countries in Eastern Europe, Asia, Africa, the Middle East and Latin America, evaluated avoidable healthcare consumption (defined as ≥1 hospitalization for cardiovascular reasons or ≥1 visit to the emergency room for any reason in the previous 12 months) in patients receiving stable lipid-lowering therapy (LLT). A total of 9049 patients (aged ≥18 years) receiving LLT for ≥3 months and who had had their low-density lipoprotein cholesterol (LDL-C) value measured on stable LLT in the previous 12 months were enrolled between August 2015 and August 2016. Patients who had received a proprotein convertase subtilisin/kexin type 9 inhibitor in the previous 6 months were excluded. Patients were stratified by cardiovascular risk level using the Systematic Coronary Risk Estimation chart for high-risk countries.

**Results:**

The proportion of patients at their LDL-C goal was 32.1% for very-high risk patients compared with 55.7 and 51.9% for patients at moderate and high cardiovascular risk, respectively. Overall, 20.1% of patients had ≥1 reported hospitalization in the previous 12 months (7.9% for cardiovascular reasons), 35.2% had ≥1 intensive care unit stay and 13.8% visited the emergency room. Avoidable healthcare resource consumption was reported for 18.7% patients overall, and in 27.8, 7.7, 7.7 and 13.2% of patients at very-high, high, moderate and low risk, respectively. Across all risk groups 22.4% of patients not at LDL-C goal and 16.6% of patients at LDL-C goal had avoidable healthcare resource consumption.

Being at very-high cardiovascular risk, having cardiovascular risk factors (including hypertension and smoking), and having factors indicating that the patient may be difficult to treat (including statin intolerance, comorbidities and chronic medication), were independent risk factors for avoidable healthcare resource consumption (all *p* <0.05).

**Conclusions:**

Healthcare resource consumption associated with adverse clinical outcomes was observed in patients on stable LLT in countries outside Western Europe and North America, particularly those at very-high cardiovascular risk and those who were difficult to treat.

## Introduction

Dyslipidemia is a major risk factor for cardiovascular disease, which is a leading cause of mortality and morbidity worldwide [1–3]. Reducing low-density lipoprotein cholesterol (LDL-C) towards recommended treatment goals is a cornerstone of dyslipidemia therapy, and lowers the risk of cardiovascular events [1, 4]. The aim of healthcare systems internationally is to deliver quality clinical care, including improvements in LDL-C management and associated clinical outcomes. However, these systems also need to be sustainable, and it is important to consider the cost-effectiveness of care, particularly in countries where healthcare resources may be limited.

Dyslipidemia is associated with increased healthcare resource utilization and costs [5–8]. This includes ‘desired’ healthcare resource consumption, eg, that associated with patient follow-up, which aims to improve patient outcomes, and ‘avoidable’ consumption of healthcare resources, resulting from clinical complications of dyslipidemia. ‘Avoidable’ consumption may be higher in patients whose management is suboptimal, ie, difficult-to-treat patients [9, 10]. Recent large-scale studies of achievement of LDL-C goals in patients with dyslipidemia have been conducted largely in Western Europe and North America [11–13], and data from other parts of the world are limited [14–17]. The International ChoLesterol management Practice Study (ICLPS) was a multinational, cross-sectional, observational study to investigate the achievement of European Society of Cardiology (ESC)/European Atherosclerosis Society (EAS) guideline LDL-C goals [1], and their determinants, in real-world clinical practice in countries outside of Western Europe and North America [18]. The primary outcome was the proportion of patients who failed to achieve their LDL-C goal according to the 2011 ESC/EAS guidelines [19]. The results of this study suggested that rates of LDL-C goal achievement were poor in some countries outside Western Europe and North America. This paper will describe healthcare resource consumption in ICLPS, one of the predefined secondary objectives of the study. The findings will add to our understanding of the impact of dyslipidemia on healthcare utilization in countries, for which data are lacking. We hypothesize that avoidable consumption of healthcare resources will be elevated in patients not at LDL-C goal.

## Methods

ICLPS was a multinational, cross-sectional, observational study conducted in 452 centers across 18 countries in Africa, Asia, Eastern Europe, Latin America and the Middle East between August 2015 and August 2016. The methods are described in detail elsewhere [18]. Achievement of LDL-C target was defined according to the 2011 ESC/EAS guidelines [19], ie, <1.8 mmol/L or 50% LDL-C reduction (for those patients whose baseline untreated LDL-C was available) when target levels could not be reached for very-high risk patients, <2.5 mmol/L or 50% LDL-C reduction for high-risk patients, and <3.0 mmol/L for moderate-risk patients. A full list of participating physician investigators is provided in the Additional file 1. The study was conducted according to the Declaration of Helsinki principles, guidelines for Good Epidemiology Practice and local regulations.

### Patients

Patients aged ≥18 years, who had been receiving a stable dose and type of lipid-lowering therapy (LLT) for ≥3 months before enrolment and had had their LDL-C value measured on stable LLT in the previous 12 months, were eligible. Proprotein convertase subtilisin/kexin type 9 (PCSK9) inhibitors were not available in all countries at the time of the study; therefore, patients who had received a PCSK9 inhibitor in the previous 6 months were excluded. All consecutive presenting patients, attending their physician for any reason, were enrolled over a predefined 2-week interval at any one site, and within a 3–6-month timeframe for each country.

Patients were stratified according to cardiovascular risk (very-high, high, moderate or low), calculated retrospectively in patients in whom the relevant data were available, using the Systematic Coronary Risk Estimation (SCORE) chart [1]. The chart for high-risk countries was selected for all participating countries as recommended in the European guidelines [1] because of the increasing rate of cardiovascular disease in non-European countries.

### Socioeconomic and healthcare consumption variables

Data on baseline demographics, residence, education, employment status, health insurance and healthcare resource consumption during the previous 12 months were collected on the patient case-report form, which was completed for each patient during a single visit. Healthcare resource consumption variables included the number of, and main reasons for, hospitalizations and visits to emergency rooms (ERs), general practitioners (GPs) and specialists (cardiovascular and other), as well as any investigations carried out (electrocardiogram [ECG], echocardiography, stress ECG and laboratory tests, including tests for cholesterol level).

### Statistical analysis

The presence of avoidable healthcare resource consumption was determined for each patient. Avoidable healthcare resource consumption was defined as at least one hospitalization for a cardiovascular reason or at least one visit to the ER. Healthcare resource consumption variables and avoidable healthcare resource consumption in the past 12 months are presented as descriptive statistics with mean ± standard deviation or median (interquartile range) for continuous variables, and as counts (percentages) for categorical data.

The factors associated with avoidable healthcare consumption were studied using two mixed categorical models: one including variables at the patient level only, and the other including variables at the patient level as well as environmental variables. Variables with a *p*-value ≤10% in univariate statistics and recorded in more than 80% of patients were checked for collinearity (with Cramer’s V statistics) and reviewed by a scientific expert (ND). Selected variables were finally proposed to the model in a descending stepwise procedure. Variables significant in the multivariable logistic regression analysis at the 5% level were retained in the final model. *C*-statistics (a measure of goodness of fit of the logistic models) were provided with the respective 95% confidence intervals. Variables tested but not significant in the multivariable logistic regression analysis at the 5% level are listed in Additional file 1: Table S1.

All authors had full access to the data presented here, and FM had access to all of the data in the study and performed the data analysis. All authors take responsibility for the study’s integrity and data accuracy.

## Results

### Patients

The demographic and socioeconomic profile of the patients is shown in Table 1. A total of 9049 patients were enrolled in the study: 9.7% from Africa, 39.2% from Asia, 9.3% from Eastern Europe, 20.8% from Latin America and 20.9% from the Middle East [18]. The mean age of the population was 60.2 ± 11.7 years and 55.0% were male (Table 1); clinically defined (by the investigating physician) hypertension was present in 71.5% and diabetes mellitus in 54.3%. SCORE cardiovascular risk could be calculated for 87.8% patients. Of these, 60.9% were at very-high risk, 33.0% were at high risk, 5.2% were at moderate risk and 0.9% were at low risk of cardiovascular disease.

**Table 1:** Demographic and socioeconomic profile of patients by risk level

	Low Risk (*n* = 70)	Moderate Risk (*n* = 411)	High Risk (*n* = 2621)	Very-High Risk (*n* = 4842)	Risk Non-Assessable (*n* = 1105)	All (*N* = 9049)
Mean (SD) age, years	37.6 (5.7)	51.8 (10.3)	57.5 (11.3)	63.3 (10.7)	57.6 (12.4)	60.2 (11.7)
Male	8 (11.4)	171 (41.6)	1152 (44.0)	3136 (64.8)	508 (46.0)	4975 (55.0)
Ethnicity	*n* = 70	*n* = 411	*n* = 2621	*n* = 4842	*n* = 1105	*n* = 9049
Asian/South Asian/Indian	34 (48.6)	163 (39.7)	1442 (55.0)	1765 (36.5)	515 (46.6)	3919 (43.3)
Black African	1 (1.4)	2 (0.5)	52 (2.0)	47 (1.0)	20 (1.8)	122 (1.3)
Caucasian/European	14 (20.0)	109 (26.5)	490 (18.7)	1829 (37.8)	232 (21.0)	2674 (29.6)
Native Latin American	5 (7.1)	34 (8.3)	256 (9.8)	357 (7.4)	144 (13.0)	796 (8.8)
Oriental/Arab/Persian	6 (8.6)	41 (10.0)	157 (6.0)	490 (10.1)	62 (5.6)	756 (8.4)
Other	10 (14.3)	62 (15.1)	224 (8.5)	354 (7.3)	132 (11.9)	782 (8.6)
Geographical region	*n* = 70	*n* = 411	*n* = 2621	*n* = 4842	*n* = 1105	*n* = 9049
Africa	1 (1.4)	20 (4.9)	266 (10.1)	504 (10.4)	90 (8.1)	881 (9.7)
Asia	23 (32.9)	135 (32.8)	1335 (50.9)	1581 (32.7)	472 (42.7)	3546 (39.2)
Eastern Europe	2 (2.9)	27 (6.6)	66 (2.5)	699 (14.4)	52 (4.7)	846 (9.3)
Latin America	19 (27.1)	140 (34.1)	554 (21.1)	837 (17.3)	336 (30.4)	1886 (20.8)
Middle East	25 (35.7)	89 (21.7)	400 (15.3)	1221 (25.2)	155 (14.0)	1890 (20.9)
Residence location	*n* = 70	*n* = 411	*n* = 2621	*n* = 4842	*n* = 1105	*n* = 9049
Urban area	60 (85.7)	351 (85.4)	2126 (81.1)	3779 (78.0)	908 (82.2)	7224 (79.8)
Rural area	3 (4.3)	32 (7.8)	252 (9.6)	515 (10.6)	99 (9.0)	901 (10.0)
Suburban area	7 (10.0)	28 (6.8)	243 (9.3)	548 (11.3)	98 (8.9)	924 (10.2)
Educational level	*n* = 70	*n* = 411	*n* = 2619	*n* = 4833	*n* = 1102	*n* = 9035
Illiterate	2 (2.9)	12 (2.9)	114 (4.4)	290 (6.0)	40 (3.6)	458 (5.1)
Primary	8 (11.4)	60 (14.6)	613 (23.4)	1125 (23.3)	200 (18.1)	2006 (22.2)
Secondary	19 (27.1)	143 (34.8)	958 (36.6)	1837 (38.0)	379 (34.4)	3336 (36.9)
University/Higher	41 (58.6)	196 (47.7)	934 (35.7)	1581 (32.7)	483 (43.8)	3235 (35.8)
Health insurance	*n* = 70	*n* = 411	*n* = 2621	*n* = 4842	*n* = 1105	*n* = 9049
National health service/ public health insurance	23 (32.9)	165 (40.1)	970 (37.0)	2781 (57.4)	452 (40.9)	4391 (48.5)
Private health insurance	16 (22.9)	130 (31.6)	457 (17.4)	657 (13.6)	234 (21.2)	1494 (16.5)
National health insurance/ public health insurance + private health insurance	2 (2.9)	12 (2.9)	95 (3.6)	240 (5.0)	24 (2.2)	373 (4.1)
No coverage	27 (38.6)	94 (22.9)	824 (31.4)	958 (19.8)	307 (27.8)	2210 (24.4)
Unknown	2 (2.9)	10 (2.4)	275 (10.5)	205 (4.2)	88 (8.0)	580 (6.4)
Insurance includes drug reimbursement	*n* = 41 29 (70.7)	*n* = 307 199 (64.8)	*n* = 1522 1005 (66.0)	*n* = 3674 2581 (70.3)	*n* = 708 426 (60.2)	*n* = 6252 4240 (67.8)
Employment status	*n* = 70	*n* = 410	*n* = 2620	*n* = 4842	*n* = 1105	*n* = 9047
Full-time	42 (60.0)	230 (56.1)	978 (37.3)	1452 (30.0)	450 (40.7)	3152 (34.8)
Part-time	10 (14.3)	25 (6.1)	131 (5.0)	230 (4.8)	52 (4.7)	448 (5.0)
Not employed/retired	18 (25.7)	155 (37.8)	1511 (57.7)	3160 (65.3)	603 (54.6)	5447 (60.2)

Data are presented as n (%) unless otherwise stated

*n*, number of patients in the sample population; *SD*, standard deviation

Most patients (79.8%) were from urban areas, and 72.7% had completed secondary education or higher. Overall, 34.8% of patients worked full-time, 5.0% worked part-time and 60.2% of patients were not employed or had retired. The majority of patients (69.1%) had healthcare insurance: 48.5% had healthcare covered by national health/public health insurance, 16.5% had private healthcare and 4.1% had both. Of the remaining patients, 24.4% had no insurance coverage and insurance status was unknown in 6.4%. Of patients with health insurance, drug reimbursement was included for 67.8%, which equates to 46.9% of all patients.

### LDL-C goal achievement

LDL-C goal achievement rates were 55.7, 51.9, and 32.1% for patients at moderate, high, and very-high cardiovascular risk, respectively [18].

### Healthcare resource consumption in the 12 months before enrolment

Table 2 and Additional file 1: Table S2 present the frequency and duration of hospitalizations in the 12 months before enrolment for all patients, by risk level and by whether or not the LDL-C goal was achieved. Overall, 20.1% of patients had at least one reported hospitalization in the 12 months before enrolment. In patients with at least one reported hospitalization, the mean hospital stay was 8.0 ± 11.3 days per hospitalization during their three most recent hospitalizations. Approximately one-third of patients (35.2%) with hospitalizations had at least one hospitalization in an intensive care unit among the previous three hospitalizations. Of patients with hospitalizations, 7.9% had at least one for cardiovascular reasons: 3.3% for myocardial infarction, 3.1% for unstable angina, 0.9% for ischemic stroke, and 2.3% for coronary revascularization. The proportion of patients with at least one reported hospitalization was 31.2% in the very-high risk group and 4.3–8.5% in the other risk groups. Corresponding values for cardiovascular hospitalizations were 14.2% and 0–0.6%, for the very-high risk group and other risk groups, respectively (Table 2). Of patients who achieved their LDL-C goal, 18.1% had at least one hospitalization (8.0% for cardiovascular reasons) compared with 24.3% (9.6%) for patients not at goal (Table 2).

**Table 2:** Frequency and duration of hospitalizations in the 12 months before enrolment by risk level and LDL-C goal achievement

	Risk Level					LDL-C Goal Achieved		All (*N* = 9049)
	Low Risk (*n* = 70)	Moderate Risk (*n* = 411)	High Risk (*n* = 2621)	Very-High Risk (*n* = 4842)	Risk Non-Assessable (*n* = 1105)	Yes (*n* = 3140)	No (*n* = 4734)	
Number (%) of hospitalizations of the patient in the last 12 months	*n* = 70	*n* = 411	*n* = 2620	*n* = 4841	*n* = 1105	*n* = 3138	*n* = 4734	*n* = 9047
At least one	3 (4.3)	31 (7.5)	177 (6.8)	1512 (31.2)	94 (8.5)	569 (18.1)	1151 (24.3)	1817 (20.1)
None	62 (88.6)	369 (89.8)	2280 (87.0)	3193 (66.0)	939 (85.0)	2417 (77.0)	3425 (72.3)	6843 (75.6)
One	3 (4.3)	27 (6.6)	150 (5.7)	1085 (22.4)	73 (6.6)	411 (13.1)	851 (18.0)	1338 (14.8)
Two	0	3 (0.7)	22 (0.8)	312 (6.4)	19 (1.7)	114 (3.6)	223 (4.7)	356 (3.9)
Three	0	1 (0.2)	3 (0.1)	81 (1.7)	1 (<0.1)	33 (1.1)	52 (1.1)	86 (1.0)
More than three	0	0	2 (<0.1)	34 (0.7)	1 (<0.1)	11 (0.4)	25 (0.5)	37 (0.4)
Unknown	5 (7.1)	11 (2.7)	163 (6.2)	136 (2.8)	72 (6.5)	152 (4.8)	158 (3.3)	387 (4.3)
At least one hospitalization in intensive care unit among the last three hospitalizations, n (%)	*n* = 3 0	*n* = 31 2 (6.5)	*n* = 177 23 (13.0)	*n* = 1508 599 (39.7)	*n* = 94 15 (16.0)	*n* = 568 228 (40.1)	*n* = 1148 396 (34.5)	*n* = 1813 639 (35.2)
At least one hospitalization for CV reasons among the last three hospitalizations, n (%)	*n* = 65 0	*n* = 400 2 (0.5)	*n* = 2458 9 (0.4)	*n* = 4708 667 (14.2)	*n* = 1033 6 (0.6)	*n* = 2989 240 (8.0)	*n* = 4577 438 (9.6)	*n* = 8664 684 (7.9)
For myocardial infarction	0	0	1 (<0.1)	286 (6.1)	2 (0.2)	116 (3.9)	171 (3.7)	289 (3.3)
For unstable angina	0	2 (0.5)	7 (0.3)	255 (5.4)	3 (0.3)	85 (2.8)	179 (3.9)	267 (3.1)
For ischemic stroke	0	0	1 (<0.1)	76 (1.6)	1 (<0.1)	21 (0.7)	56 (1.2)	78 (0.9)
For coronary revascularization	0	0	2 (<0.1)	196 (4.2)	1 (<0.1)	70 (2.3)	128 (2.8)	199 (2.3)
Durations (days) of last three hospitalizations^a^	*n* = 3	*n* = 30	*n* = 176	*n* = 1471	*n* = 92	*n* = 557	*n* = 1120	*n* = 1772
Mean ± SD	3.7 ± 3.1	8.4 ± 4.5	6.5 ± 6.2	8.2 ± 12.1	7.6 ± 6.3	8.7 ± 17.6	7.7 ± 6.8	8.0 ± 11.3
Median (IQR)	3.0 (1.0–7.0)	9.5 (4.0–11.0)	4.0 (2.0–10.0)	6.0 (3.0–10.0)	6.0 (2.2–10.5)	6.0 (3.0–10.0)	6.0 (3.0–10.0)	6.0 (3.0–10.0)
Durations (days) of last three hospitalizations^a^								
For myocardial infarction	–	–	*n* = 1	*n* = 275	*n* = 1	*n* = 112	*n* = 164	*n* = 277
Mean ± SD			23.0	7.3 ± 5.6	1.0	7.7 ± 5.5	7.1 ± 5.9	7.3 ± 5.7
Median (IQR)	–	–	23.0 (23.0–23.0)	5.0 (3.0–9.0)	1.0 (1.0–1.0)	7.0 (3.0–10.0)	5.0 (3.5–8.0)	5.0 (3.0–9.0)
For unstable angina	–	*n* = 2.0	*n* = 7	*n* = 254	*n* = 3	*n* = 85	*n* = 178	*n* = 266
Mean ± SD	–	3.0 ± 0.0	6.7 ± 7.3	6.4 ± 4.6	8.3 ± 5.9	6.1 ± 4.6	6.5 ± 4.6	6.4 ± 4.6
Median (IQR)	–	3.0 (3.0–3.0)	4.0 (2.0–12.0)	5.0 (3.0–10.0)	6.0 (4.0–15.0)	4.5 (3.0–8.0)	5.0 (3.0–10.0)	5.0 (3.0–10.0)
For ischemic stroke	–	–	*n* = 1	*n* = 76	*n* = 1	*n* = 21	*n* = 56	*n* = 78
Mean ± SD	–	–	1.0 (0.0)	10.4 ± 12.9	15.0 ± 0.0	11.6 ± 21.4	9.8 ± 7.8	10.3 ± 12.8
Median (IQR)	–	–	1.0 (1.0–1.0)	7.5 (5.0–12.0)	15.0 (15.0–15.0)	6.0 (4.0–11.0)	8.0 (5.0–13.25)	7.5 (5.0–12.0)
For coronary revascularization	–	–	*n* = 2	*n* = 194	*n* = 1	*n* = 70	*n* = 126	*n* = 197
Mean ± SD	–	–	1.5 ± 0.7	5.6 ± 5.4	1.0	5.9 ± 6.2	5.4 ± 4.8	5.6 ± 5.4
Median (IQR)	–	–	1.5 (1.0–2.0)	4.0 (2.0–7.0)	1.0 (1.0–1.0)	3.5 (2.0–8.0)	4.0 (2.0–6.0)	4.0 (2.0–7.0)

^a^Mean of last three hospitalizations

*CV*, cardiovascular; *IQR*, interquartile range; *LDL-C*, low-density lipoprotein cholesterol; *n*, number of patients in the sample population; *SD*, standard deviation

The number of visits to ERs, GPs and specialists, and the number of tests performed in the 12 months before enrolment are shown in Table 3. Patients visited an ER a mean of 0.32 ± 1.11 times. The mean number of visits to GPs, cardiovascular specialists and other specialists, was 2.71 ± 3.87, 1.83 ± 2.14 and 2.49 ± 4.30, respectively. Patients underwent a mean of 1.57 ± 2.16 ECGs, 0.56 ± 0.81 echocardiography tests and 0.18 ± 0.45 stress ECGs. For 98.3% of the patients, the investigator noted a laboratory test that included cholesterol in the 12 months before enrolment (2.10 ± 1.45 tests); however, it should be noted that the inclusion criteria of the study required all patients to have had at least one LDL-C value measured in the previous 12 months. In the very-high risk group, 18.6% of patients had at least one visit to an ER compared with 7.6–13.2% of patients in other risk groups (Table 3).

**Table 3:** Number of visits to ERs, GPs and specialists, number of tests, and avoidable healthcare resource consumption in the 12 months before enrolment by risk level and LDL-C goal achievement

	Risk Level					LDL-C Goal Achieved		All (*N* = 9049)
	Low Risk (*n* = 70)	Moderate Risk (*n* = 411)	High Risk (*n* = 2621)	Very-High Risk (*n* = 4842)	Risk Non-Assessable (*n* = 1105)	Yes (*n* = 3140)	No (*n* = 4734)	
At least one visit to the ER in the last 12 months, n (%)	*n* = 68 9 (13.2)	*n* = 395 30 (7.6)	*n* = 2440 194 (8.0)	*n* = 4649 865 (18.6)	*n* = 1053 89 (8.5)	*n* = 2955 353 (11.9)	*n* = 4529 736 (16.3)	*n* = 8605 1187 (13.8)
Number of visits to:								
An ER	*n* = 52	*n* = 319	*n* = 1863	*n* = 3773	*n* = 820	*n* = 2355	*n* = 3600	*n* = 6827
Mean ± SD	0.23 ± 0.55	0.21 ± 0.82	0.19 ± 0.75	0.42 ± 1.32	0.19 ± 0.78	0.26 ± 0.92	0.39 ± 1.28	0.32 ± 1.11
Median (IQR)	0.0 (0.0–0.0)	0.0 (0.0–0.0)	0.0 (0.0–0.0)	0.0 (0.0–0.0)	0.0 (0.0–0.0)	0.0 (0.0–0.0)	0.0 (0.0–0.0)	0.0 (0.0–0.0)
A GP	*n* = 57	*n* = 330	*n* = 2017	*n* = 3747	*n* = 902	*n* = 2438	*n* = 3656	*n* = 7053
Mean ± SD	2.54 ± 2.40	2.78 ± 3.89	2.91 ± 3.34	2.66 ± 4.30	2.44 ± 3.06	2.66 ± 3.69	2.81 ± 4.17	2.71 ± 3.87
Median (IQR)	2.0 (0.0–4.0)	2.0 (0.0–4.0)	2.0 (0.0–4.0)	1.0 (0.0–4.0)	2.0 (0.0–3.0)	2.0 (0.0–4.0)	2.0 (0.0–4.0)	2.0 (0.0–4.0)
A CV specialist	*n* = 55	*n* = 345	*n* = 1992	*n* = 4402	*n* = 870	*n* = 2630	*n* = 4109	*n* = 7664
Mean ± SD	1.05 ± 1.63	1.41 ± 1.71	0.99 ± 1.53	2.37 ± 2.32	1.30 ± 1.77	1.92 ± 2.30	1.90 ± 2.10	1.83 ± 2.14
Median (IQR)	0.0 (0.0–2.0)	1.0 (0.0–2.0)	0.0 (0.0–2.0)	2.0 (1.0–3.0)	1.0 (0.0–2.0)	1.0 (0.0–3.0)	2.0 (0.0–3.0)	1.0 (0.0–3.0)
Another specialist	*n* = 58	*n* = 337	*n* = 1991	*n* = 3845	*n* = 800	*n* = 2441	*n* = 3732	*n* = 7031
Mean ± SD	2.81 ± 4.59	2.12 ± 3.78	2.15 ± 3.47	2.91 ± 4.93	1.38 ± 2.44	2.74 ± 4.72	2.55 ± 4.29	2.49 ± 4.30
Median (IQR)	2.0 (0.0–3.0)	1.0 (0.0–3.0)	1.0 (0.0–3.0)	1.0 (0.0–4.0)	0.0 (0.0–2.0)	1.0 (0.0–3.0)	1.0 (0.0–3.0)	1.0 (0.0–3.0)
Number of tests								
ECG	*n* = 62	*n* = 352	*n* = 2125	*n* = 4379	*n* = 927	*n* = 2678	*n* = 4178	*n* = 7845
Mean ± SD	0.92 ± 0.84	0.88 ± 0.95	1.08 ± 1.21	1.98 ± 2.62	1.08 ± 1.15	1.58 ± 2.52	1.69 ± 2.07	1.57 ± 2.16
Median (IQR)	1.0 (0.0–1.0)	1.0 (0.0–1.0)	1.0 (0.0–1.0)	1.0 (0.0–3.0)	1.0 (0.0–1.0)	1.0 (0.0–2.0)	1.0 (1.0–2.0)	1.0 (0.0–2.0)
Echocardiography	*n* = 55	*n* = 334	*n* = 1949	*n* = 4218	*n* = 848	*n* = 2549	*n* = 3952	*n* = 7404
Mean ± SD	0.29 ± 0.46	0.34 ± 0.53	0.29 ± 0.56	0.75 ± 0.91	0.34 ± 0.60	0.53 ± 0.79	0.63 ± 0.85	0.56 ± 0.81
Median (IQR)	0.0 (0.0–1.0)	0.0 (0.0–1.0)	0.0 (0.0–0.0)	1.0 (0.0–1.0)	0.0 (0.0–1.0)	0.0 (0.0–1.0)	0.0 (0.0–1.0)	0.0 (0.0–1.0)
Stress ECG	*n* = 54	*n* = 323	*n* = 1914	*n* = 3857	*n* = 827	*n* = 2396	*n* = 3698	*n* = 6975
Mean ± SD	0.15 ± 0.36	0.18 ± 0.40	0.10 ± 0.32	0.22 ± 0.51	0.15 ± 0.39	0.17 ± 0.44	0.19 ± 0.47	0.18 ± 0.45
Median (IQR)	0.0 (0.0–0.0)	0.0 (0.0–0.0)	0.0 (0.0–0.0)	0.0 (0.0–0.0)	0.0 (0.0–0.0)	0.0 (0.0–0.0)	0.0 (0.0–0.0)	0.0 (0.0–0.0)
Lab test including cholesterol	*n* = 68	*n* = 403	*n* = 2545	*n* = 4695	*n* = 1062	*n* = 3053	*n* = 4590	*n* = 8773
Mean ± SD	2.41 ± 1.35	2.26 ± 1.18	2.06 ± 1.29	2.16 ± 1.62	1.86 ± 1.03	2.13 ± 1.58	2.14 ± 1.44	2.10 ± 1.45
Median (IQR)	2.0 (2.0–3.0)	2.0 (1.0–3.0)	2.0 (1.0–2.0)	2.0 (1.0–3.0)	2.0 (1.0–2.0)	2.0 (1.0–3.0)	2.0 (1.0–3.0)	2.0 (1.0–3.0)
Avoidable healthcare consumption^a^, *n* (%)	*n* = 68 9 (13.2)	*n* = 405 31 (7.7)	*n* = 2584 200 (7.7)	*n* = 4799 1332 (27.8)	*n* = 1073 94 (8.8)	*n* = 3099 515 (16.6)	*n* = 4689 1048 (22.4)	*n* = 8929 1666 (18.7)

^a^Defined as at least one hospitalization for CV reasons or at least one visit to the ER

*CV*, cardiovascular; *ECG*, electrocardiogram; *ER*, emergency room; *GP*, general practitioner; *IQR*, interquartile range; *LDL-C*, low-density lipoprotein cholesterol; *n*, number of patients in the sample population; *SD*, standard deviation

### Avoidable healthcare resource consumption

Across all patients, 18.7% had avoidable healthcare resource consumption in the previous 12 months (Table 3). This was 27.8% in patients in the very-high risk group compared with 7.7–13.2% in patients in other risk groups, and 22.4% in patients not at LDL-C goal compared with 16.6% in patients at LDL-C goal.

**Fig. 1:**
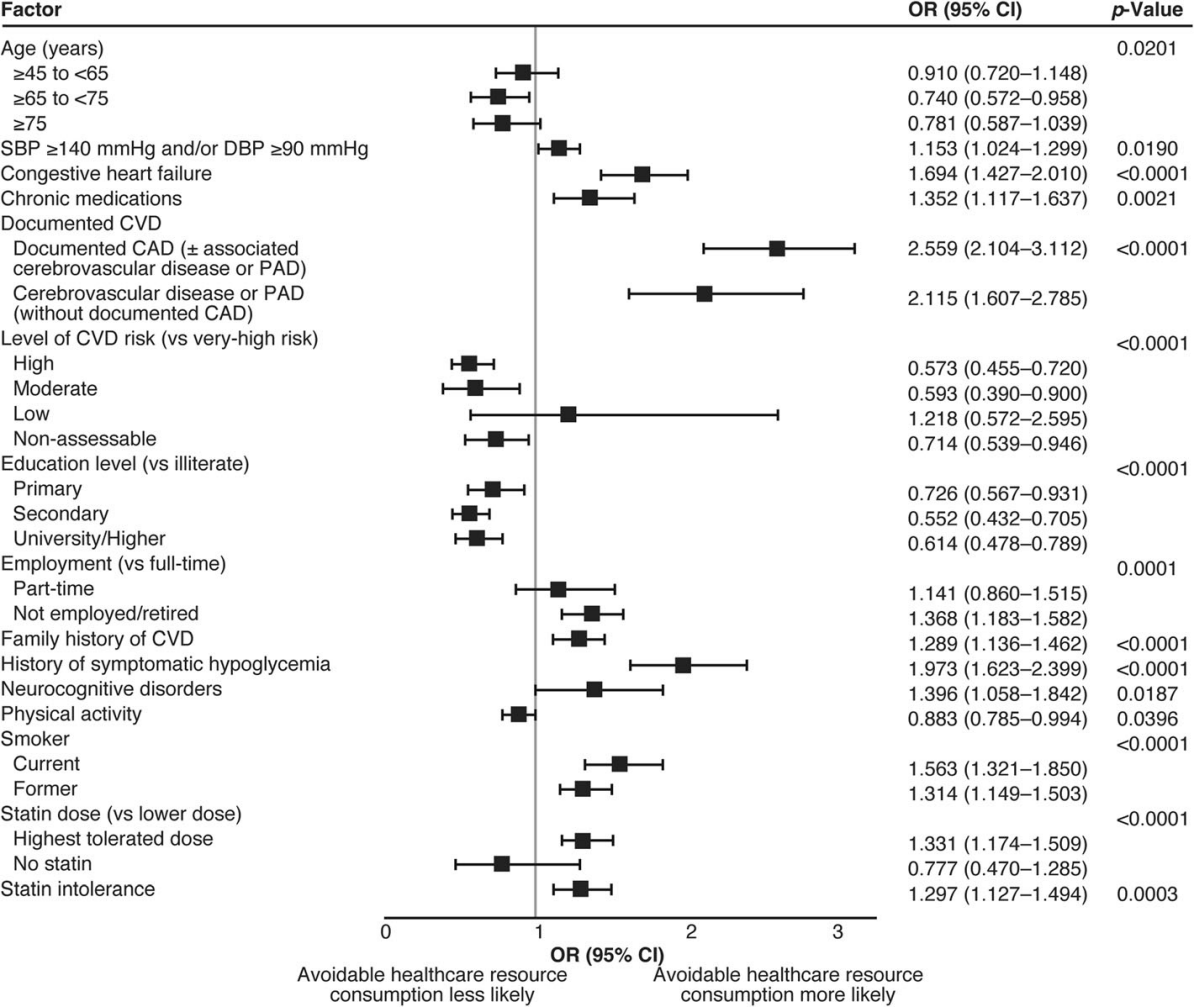
Factors independently associated with avoidable healthcare consumption (mixed model of variables at patient level only; *C*-statistic: 0.7454 [0.7329; 0.7580]). CAD, coronary artery disease; CI, confidence interval; CVD, cardiovascular disease; DBP, diastolic blood pressure; OR, odds ratio; PAD, peripheral artery disease; SBP, systolic blood pressure

**Fig. 2:**
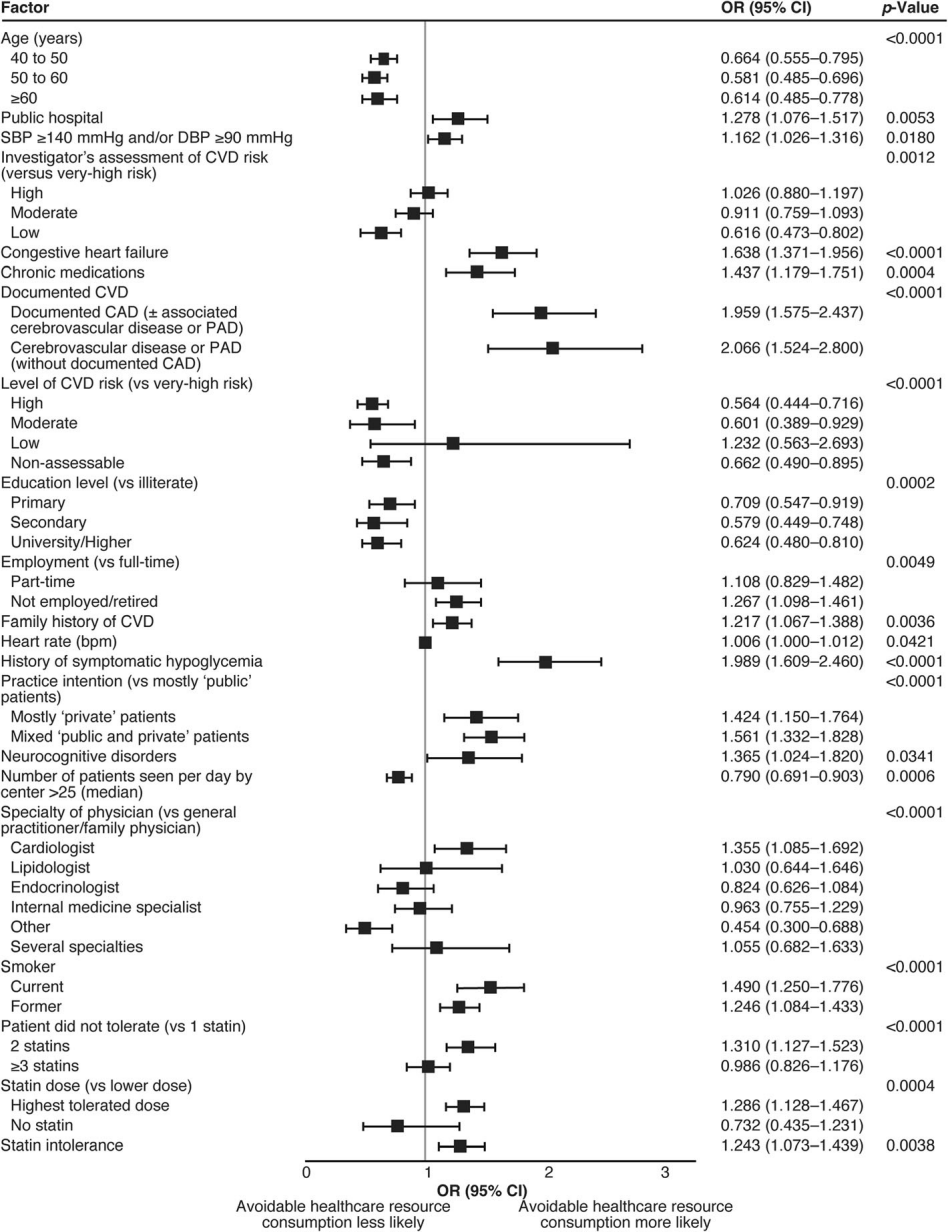
Factors independently associated with avoidable consumption of healthcare resources (mixed model of variables at patient level and environmental variables; *C*-statistic: 0.7591 [0.7466; 0.7715]). CAD, coronary artery disease; CI, confidence interval; CVD, cardiovascular disease; DBP, diastolic blood pressure; OR, odds ratio; PAD, peripheral artery disease; SBP, systolic blood pressure

The findings of the multivariable analysis of avoidable healthcare resource consumption based on variables at the patient level only are shown in Fig. 1. Younger age, hypertension, congestive heart failure, chronic medication, documented cardiovascular disease, family history of cardiovascular disease, history of hypoglycemia, neurocognitive disorders, smoking, being on the highest tolerated dose of statin and statin intolerance were all independently associated with avoidable healthcare resource consumption. Moderate and high versus very-high cardiovascular risk, being educated, full-time employment and being physically active were associated with decreased avoidable healthcare resource consumption. When the multivariable analysis was performed using variables at the patient level and environmental variables (Fig. 2), the following additional independent risk factors for avoidable healthcare resource consumption were identified: the enrolling physician was in a public hospital, the patient had increased heart rate, the practice treated private (mostly private or mixed public and private) patients, the enrolling physician was a cardiologist and the patient had intolerance to two statins. Additional factors associated with a decrease in avoidable healthcare resource consumption were an investigator’s assessment (based on their clinical judgement) of patient’s risk as low, the practice was seeing >25 patients per day, and the physician having a specialty of ‘other’.

The proportion of patients with avoidable healthcare resource consumption differed by type of health insurance (none, 14%; public, 22.6%; private, 16.3%; both public and private, 16.3%; *p* ≤0.001) when tested using univariate statistics. However, this effect was not retained in the multivariate models (Additional file 1: Table S1).

## Discussion

In this multinational, cross-sectional, observational study, avoidable healthcare resource consumption was observed in 18.7% of patients on stable LLT in countries outside Western Europe and North America (16.6% of patients at LDL-C goal; 22.4% of patients not at goal), reflecting a high rate of adverse clinical outcomes among this population. Avoidable healthcare resource consumption occurred in 27.8% of patients at very-high cardiovascular risk, at least double the proportion of patients at lower risk. LDL-C goal achievement rate in the very-high risk group was particularly low (32.1% vs 55.7 and 51.9% for patients at moderate and high risk, respectively). Factors that may contribute to this low LDL-C goal achievement rate in very-high risk patients include suboptimal use of LLTs [18], more stringent LDL-C goals (ie, <70 mg/dL) and either failure of physicians to adhere to them in this group or a lack of efficacy of currently used LLTs for achieving these goals. Previous studies in Europe and the USA have reported an increase in healthcare resource utilization associated with cardiovascular outcomes in patients with dyslipidemia that persists for several years after an acute cardiovascular event [6, 7]. This healthcare burden may be decreased by effective management of LDL-C levels to prevent cardiovascular events [8].

The findings of the multivariable analyses suggested several factors independently associated with avoidable healthcare resource consumption in this patient population. Avoidable healthcare resource consumption was as least twice as high in patients with than without documented cardiovascular disease. It was also higher in patients with other cardiovascular risk factors, including elevated blood pressure, smoking, history of symptomatic hypoglycemia and family history of cardiovascular disease. The presence of congestive heart failure, neurocognitive disorders, the use of chronic medications, taking the highest tolerated dose of statin or being statin intolerant were also associated with higher avoidable healthcare resource consumption. Although we cannot draw a conclusion on a formal causal relationship because of the study design, these data may reflect a higher rate of complications in difficult-to-treat patients, ie, those who cannot tolerate effective doses of statins, whose dyslipidemia is resistant to statins, and whose physicians may be reluctant to treat aggressively due to the presence of comorbidities and/or the use of concomitant therapies. These findings are consistent with the results of other studies that have investigated healthcare resource consumption in difficult-to-treat patients. For example, a recent US observational study of data from an integrated health system’s electronic health records between 2008 and 2014 compared outcomes between statin-intolerant patients and matched controls (statin-treated patients matched for age, sex, cardiovascular risk category, comorbidities and concomitant medications using propensity score matching) [9]. Patients with statin intolerance were less likely to reach LDL-C goals, incurred higher healthcare costs and experienced a higher rate of adverse cardiovascular events compared with controls. Another retrospective cohort study demonstrated a significantly higher frequency of visits to the ER, and higher total healthcare costs, in patients with low adherence to statin therapy (defined as medication possession ratio <40%) over a 1-year follow-up period after treatment initiation [10].

Patient factors associated with lower avoidable healthcare resource consumption, such as being educated, in full-time employment and physically active, may indicate a greater ability or commitment of the patient to managing their dyslipidemia. The influence of physician factors on avoidable healthcare resource consumption likely results from variations in the severity of patients seen by different specialties of physicians, the practice’s level of experience with dyslipidemia, and differences in the availability of resources between the public and private healthcare sectors. Low cardiovascular risk assessed by SCORE was associated with higher avoidable healthcare resource consumption, whereas low cardiovascular risk assessed by the investigator was associated with lower avoidable healthcare resource consumption. This difference may reflect the low agreement between SCORE and investigator-assessed risk level in the study [18], and highlights the importance of accurate risk assessment to optimize LDL-C management and improve clinical outcomes.

### Limitations

This study was subject to limitations that may have influenced its findings; these have been outlined previously [18]. Of note here is the cross-sectional nature of the study, which does not allow investigation of causal effects. Furthermore, the study population is not fully representative of all patients treated with LLT in each country. The patients were mainly educated urban residents with health insurance, and as a result were likely to have better access to healthcare than the general population. Consequently, the results may overestimate both LDL-C goal achievement and healthcare resource consumption in these countries. The study population is heterogenous being enrolled from countries with different healthcare systems and cardiovascular risks. Africa and Eastern Europe were underrepresented in the study population. In addition, recording of healthcare resource consumption data by investigators was not always complete; therefore, missing data may also have influenced the interpretation of results. As avoidable healthcare resource consumption was defined as ≥1 hospitalization for cardiovascular reasons or ≥1 visit to the emergency room for any reason in the previous 12 months, it may have included some healthcare resource consumption related to causes other than dyslipidemia. Finally, as healthcare resource consumption was studied in the year preceding study entry but the duration of LLT varied between patients (inclusion criterion: >3 months prior to enrolment) it cannot be ruled out that the clinical events leading to increased healthcare resource consumption may have influenced achievement of LDL-C goals thereafter. It would be likely, however, that events such as acute coronary syndromes would have led to optimization of LLT, rather than the reverse.

## Conclusions

Avoidable healthcare resource consumption was observed in approximately one-fifth of patients on stable LLT in countries outside Western Europe and North America, and in more than one-quarter of patients at very-high cardiovascular risk. Being at very-high cardiovascular risk, having cardiovascular risk factors and having factors indicating difficult-to-treat patients, were independently associated with an increased avoidable healthcare consumption.

## Supplementary information

**Additional file 1:.**

## Data Availability

Qualified researchers may request access to patient level data and related study documents including the clinical study report, study protocol with any amendments, blank case report form, statistical analysis plan and dataset specifications. Patient level data will be anonymized and study documents will be redacted to protect the privacy of trial participants. Further details on Sanofi’s data sharing criteria, eligible studies and process for requesting access can be found at: https://www.clinicalstudydatarequest.com

## Abbreviations

CAD: Coronary artery disease
CI: Confidence interval
CV: Cardiovascular
CVD: cardiovascular disease
DBP: Diastolic blood pressure
ECG: Electrocardiogram
ESC: European Society of Cardiology
EAS: European Atherosclerosis Society
ER: Emergency room
GP: General practitioner
ICLPS: International ChoLesterol management Practice Study
IQR: Interquartile range
LDL-C: Low-density lipoprotein cholesterol
LLT: Lipid-lowering therapy
n: Number of patients in the sample population
OR: Odds ratio
PAD: Peripheral artery disease
PCSK9: Proprotein convertase subtilisin/kexin type 9
SBP: Systolic blood pressure
SCORE: Systematic Coronary Risk Estimation
SD: Standard deviation
