# Supplementary material for: Healthcare resource utilization in patients on lipid-lowering therapies outside Western Europe and North America: findings of the cross-sectional observational International ChoLesterol management Practice Study (ICLPS)
Source: Lipids Health Dis. 2020 Apr 7;19:64. doi: 10.1186/s12944-020-01235-5 (PMC7140553; doi:10.1186/s12944-020-01235-5)
Supplement: Supplementary file 1 — Additional file 1:. [file 12944_2020_1235_MOESM1_ESM.docx]

SUPPLEMENTARY APPENDIX

## Contents

[List of International ChoLesterol management Practice Study (ICLPS) investigators and patient enrollment numbers 2](#_Toc3884060)

[Investigators who enrolled at least one patient 3](#_Toc3884061)

[Supplementary Table 1. Duration of hospitalizations in the 12 months before enrollment by risk level and LDL-C goal achievement (imputing 0 if no hospitalization) 9](#_Toc3884062)

# List of International ChoLesterol management Practice Study (ICLPS) investigators and patient enrollment numbers

| **Region  (no. of patients enrolled, %)** | **Country** | **No. patients enrolled** | **Principal Investigator/National Coordinator** |
| --- | --- | --- | --- |
| Eastern Europe  (*n* = 846, 9.3%) | Russia | 349 | Yuri Karpov (Principal Investigator and National Coordinator), Russian Cardiology Research & Development Complex, Moscow, Russia |
|  | Ukraine | 497 | Olena Mitchenko (National Coordinator), National Scientific Center: “M.D. Strazhesko Institute of Cardiology” Academy of Medical Sciences of Ukraine, Narodnogo Opolcheniya Kyiv, Ukraine |
| Asia  (*n* = 3546, 39.2%) | Bangladesh | 499 | Abdul Wadud Chowdhury (Principal Investigator and National Coordinator), Dhaka Medical College Hospital, Dhaka, Bangladesh |
|  | India | 2013 | Kaul Upendra (Principal Investigator and National Coordinator), Fortis Escorts Heart Institute & Research Centre, Fortis Hospitals, New Delhi, India |
|  | South Korea | 1034 | Kim Sung Rae (National Coordinator), Bucheon St. Mary’s Hospital, Wonmi-gu, Bucheon-si, Gyeonggi-do, Republic of Korea.  Choi Sung-Hee (National Coordinator), Seoul National University Bundang Hospital, Bundang‑gu, Seongnam-si, Gyeonggi-do, Republic of Korea |
| Africa  (*n* = 881, 9.7%) | Algeria | 485 | Abdelkrim Berrah (National Coordinator), Service de Médecine Interne, Centre Hospitalo-Universitaire, Mohamed Lamine Debaghine Bab El Oued, 16000 Algiers, Algeria |
|  | South Africa | 396 | Dirk Blom (Principal Investigator and National Coordinator), Lipid Laboratory, University of Cape Town, Cape Town, South Africa |
| Middle East (*n* = 1890, 20.9%) | Israel | 99 | Yossi Azuri (Principal Investigator and National Coordinator), Maccabi Health Care Services, Tel Aviv, Israel |
|  | Kuwait | 150 | Mohammed Zubaid (Principal Investigator and National Coordinator), Mubarak Hospital, Jabriya, Kuwait |
|  | Oman | 97 | Khalid Al Rasahdi (Principal Investigator and National Coordinator), Sultan Qaboos University, Muscat, Oman |
|  | Lebanon | 200 | Rabih Azar (Principal Investigator and National Coordinator), Hotel Dieu De France Hospital, Rue al Syrian, Ashrafieh, Beirut, Lebanon |
|  | Saudi Arabia | 231 | Mohammed Balgaith (Principal Investigator and National Coordinator), Cardiology Center, National Guard Hospital, Riyadh, Saudi Arabia |
|  | Turkey | 830 | Meral Kayıkçıoğlu (Principal Investigator and National Coordinator), Ege University Faculty of Medicine Department of Cardiology, Bornova, İzmir, Turkey |
|  | United Arab Emirates | 283 | Wael Mahameed (National Coordinator), Sheikh Khalifa Medical City, Abu Dhabi, United Arab Emirates |
| Latin America (*n* = 1886, 20.8%) | Argentina | 307 | Carlos Alberto Cuneo (Principal Investigator and National Coordinator), Prevencion Cardiovascular Salta, Provincia de Salta, Argentina |
|  | Brazil | 499 | Raul Santos (Principal Investigator and National Coordinator), Instituto Do Coração Do Hospital Das Clínicas Da Faculdade De Medicina Da Universidade De São Paulo, São Paulo, Brazil |
|  | Colombia | 454 | Alvaro J. Ruiz (National Coordinator), San Ignacio Hospital, Pontificia Universidad Javeriana, Bogotá, Colombia |
|  | Mexico | 626 | Carlos A. Aguilar Salinas (Principal Investigator), Instituto Nacional de Ciencias Medicas y Nutricion, Mexico City, Mexico |
| Total population |  | 9049 |  |

## Investigators who enrolled at least one patient

**Algeria:** Hadjissa Khadidja, Zebbar Nacerddine, Gouader Moussa, Senouci Fatima, Benissad Houria, Tazdait Rafik, Hamidouche Karima, Ayache Ahmed, Achaibou Rachid, Dahmane Saida, Benkhodja Mohamed Bachir, Louaifi Ali, Remili Rachid, Benbouabdellah Belkacem, Hadjkali Abdelhamid, Manseur Fahima, Bertal Sabra Amina, Methia Nadira, Kadour Fatima, Latreche Samia, Agrane Khadidja, Menzou Farouk, Kichou Brahim, Djellaoudji Azzouz, Khelil Saida, Ghemri Sofiane, Nibouche Djamel Eddine, Djeghri Nora, Belguedj Rinda, AbdelBaki Mourad, Kachenoura Aldjiia, Krim Messaad, Belkadi Zahoua, Henine Nora, Aoudia Yazid, Aouiche Samir, Yakhou Mohamed, Belhadj Fatima, Merdjana Karima, Ziani Samia, Benatmane Houria, Zellat Khiera, Faraoun Khadra, Douar Malika, Djafri Yasmina, Bouamrane Nadia, Cherief Fadila, Khellaf Hadda, Zidani Hocine, Baghous Houssem.

**Argentina:** Carlos Alberto Cuneo, Gabriel Dario Waisman, Jorge Roberto Aiub, Alejandro Hershson, Carol Kotliar, Julio Andres Vallejos, Juan Carlos Medrano, Alberto Juan Lorenzatti, Alfredo Lozada, Pablo Corral.

**Bangladesh:** Chowdhury Abdul Wadud, Haque Azizul, Chakraborti Rajashish, Hussain Md. Tofazzal, Reza Salim, Uddin Mir Jamal, Amin Mohammad Gaffar, Islam Abul Hasan Md. Waliul, Rahman Md. Toufiqur, Akter Mohammed Shamim, Khan Kaiser Nasrullah, Hossain Tanjina, Prasad Indrajit, Amin Ahsanul Haque, Ishaque SM, Habib Maruf Bin, Ali Hassan Kazi, Rabbani Raihan, Bhowmik Nirmalendu Bikash, Hassan Mohammad Nabiul.

**Brazil:** Dias Raul, Izar Maria Cristina, Saporito Wladimir, Cesena Fernando, Saraiva Jose, Maia Lilia, Ardito Wilma, Ayoub Jose, Manenti Euler, Fontana Hugo, Moraes João, Hissa Miguel, Pedrosa Hermelinda, Antunes Daniela, Lisboa Hugo, Oigman Willie, Silva Antonio, Lima Sonia, Isaia Carlos, Giraldez Viviane, Rossi Fabio.

**Colombia:** Puerta Carlos Felipe, Rincon Nelson Andres, Quintero Stella, Fandiño Alberto, Abaunza Leonidas, Jacome Andres Mauricio, Lenis Claudia Patricia, Lossa Claudia, Holguin Diego, Orozco Liliana, García Olga Lucia, Balaguera Jose Francisco, Cohen Luz Helena, Sotomayor Aristides, Jaramillo Carlos Francisco, Murillo Nelson, Lievano Manuel, Vesga Boris, Quintero Adalberto, Pantoja Dolly, Chacon Maria del Pilar, Lujan Dilcia Maria, Yepes Carlos Augusto, Lopez Claudia Tatiana, Yupanqui Hernan, Botero José Fernando, Yepes Emerson, Ibarra Jaime, Rueda Javier Enrique, Florez Jose Luis, Molina Dora, Coronel Julian.

**India:** Kaul Upendra, Krishnamurthy Deepak, Chandra Subash, Reddy Y Vijaya Chandra, Chopda Manoj, Sinha Sudhir Chandra, Kesavamurthy C B, Khan Idris Ahmed, Dave Tarun, Kawthekar Girish, Chopra Vijay Kumar, Kothari Dipesh, Kumar Ravi, Singh Kiran Pal, Ballani Piya, Modi KD, Sanyal Debmalya, Prabhu Mukhyaprana, Nagendar Jakka, Sharma Surender Kumar, Patnala Sreedevi, Shah Jayesh, Shah Siddharth, Roy Amitabha, Jain Gaurav, Sanghvi MD, Mutha Abhay, Dani Praveen, Madhavan R, Gupta JB, Bhattacharyya Paramartha, Somani Vinod, Rohatgi Gyanendra Mohan, Sanghvi Snehal, Vasudevan Sujit, Verma Sachin, Ganga Kiran, Gupta Rajeev, Verma Sanjay, Gangakhed Satish Kumar, Beswal Gaurav, J Ramdas, Mallikarjuna Rao KV, Vadavi Arun, Marya Rajesh, Mathew Joy, Rau Ram Mohan, Manu Sharma, Sonawane Sandeep, Ravindra Sethiya, Gandhi Sanjay, Mathew S, Kalanitthi A, Shah Ashok, Mahajan Sanjay, Pathak HS, Julka Sandeep, Gupta Sanjay, Nagre Santosh, Dhand Sunil, Bannerjee Abhijeet, T Madana Mohan, Haldar Susovan, George Abraham P, Agarwal Sanjay, Gheewala Nalin, Jain Pradeep Kumar, Tewari Ajoy K, Baldawa Vishnu, Kumar JS, Khanna Monica, Mathai Johnson, Rao Sanjay, Poddar Mahesh, Mane Rajendra, Sethia Ashok, Rao Visweswar, Perumal Nalla, Kumar J Aman, Kongara Srikanth, Hari K, Tongaonkar Aniruddha, Gupta Sanjeev, Marda Mahesh, Jain Sunil, Sethi Kamal Kumar, Razzak MA, Rao Dayasagar, Dhar Pramod Kumar, Shah Devang.

**Israel:** Orlovsky Sophia, Chornia Larisa, Ciuraru George, Feldman Arie, Doctor Ruth, Eliyahu Vered, Radzinski Irena, Pomeranz Avishalom, Golzman Boris, Geva Adi.

**Republic of Korea:** Sung-Il Sohn, Sung-Hee Choi, Yong-Jin Kim, Seung-Jin Oh, Jin-Ho Shin, Bo-Hyun Kim, Woo-Keun Seo, Jae-Kwan Cha, Kyoung-Im Cho, Hye-Soon Kim, Jin-Won Kim, Sang‐Chol Lee, Shin-Jae Kim, Sang-Yong Kim, Jung-Rae Cho, Jung-Sun Kim, Kyung-Mook Choi, Soon-Hee Lee, Kang-Wook Lee.

**Kuwait:** Zubaid Mohammed, Farrag Ebrahim, Saad Hisham, Abd Al Monem Amro, Hasan Ebrahim, Mekhaeil Medhat, Lashin Ebrahim.

**Lebanon:** Mouawad Walid, Keshishian Sahak, Amm Mireille, Andari Emile, Zind Romel, Ballout Hajar, Mohamad Malek, Moukheiber Sami, Abdel Massih Tony, Azar Rabih.

**Mexico:** César Gonzalo Calvo Vargas, Edmundo Bayram llamas, Esperanza Martinez Abundis, Gerardo Andres Baez Vargas, Pedro Mendoza Martinez, Rodrigo Navarrete Valencia, Bernardo Emilio Valenzuela Salazar, Francisco Javier Robledo Gutierrez, Alfredo Nacoud Askar, Carlos Alberto Aguilar Salinas, Sergio Zuñiga Guajardo, Maria Elena Cedano Limon, Roberto Bejarano Rodriguez, Lirio de Maria Delgado Garcia, Juan Carlos Villanueva Arias, Lucia Alejandra Castillo Vigna, José Gerardo Gonzalez Gonzalez, Martha Leticia López Velazco.

**Oman:** Khalid Al Rasahdi, Khalid Al Wali, Mustafa Al Hinai, Ali Mamari, Omayma Elshafaie, Mehar Ali.

**Russia:** Karpov Yuri, Ezhov Marat, Khaisheva Larisa, Lyamina Nadezhda, Viktorova Inna, Kosmacheva Elena, Khasanov Niyaz, Libis Roman, Tretyakova Tatyana, Koziolova Natalya, Eliseeva Liudmila, Napalkov Dmitriy, Karetnikova Victoriya, Boytsov Sergey.

**Saudi Arabia:** Shukri Al Sail, Osama El Khateeb, Maryam Al Qaseer, Rashid Al Jawair, Eman Ashgar, Abdulwahab Bawahab, Hassan El Sayed, Mohammed Balghith.

**South Africa:** Blom Dirk, Amod Aslam, Chelin Neville, Coetzee Kathleen, Corbett Clive, Fouche Leon, Fourie Nyda, Govind Uttam, Joshi Shaifali, Kapp Cornelia, Kotze Hester, Makan Hemant, Mahomed Akbar, Ngcakani Nomangesi Judith, Padayachee Trevenesan, Raal Frederick, Seedat Saadiya, Snyman Hans, Trokis Julien.

**United Arab Emirates:** Azan Binbrek, Ali Aslani, Yasser El Henawy, Arshad Rasheed, Mouna Mohamdioua Boukhanera, Guevarra Daffodills, Kais Ismail Mrabet, Fahad Baslaib, Vani Krishna, Jaison T M, Nooshin Bazargani, Hashemi Pour, Katherine Rose, Lalit Maheshwari.

**Turkey:** Kayıkçıoğlu Meral, Akbulut Mehmet, Arık Osman Ziya, Ural Dilek, Yılmaz Remzi, Öner Abdülvedat, Karahan Burhan, Örem Cihan, Temizhan Ahmet, Erdoğu İsmail, Kemaloğlu Melek Didem, Kırma Cevat, Altunkeser Bülent Behlül, Gökçe Mustafa, Şabanoğlu Cengiz, Aksoy Mehmet, Şahin Mahmut, Taştan Ahmet, Keser Ahmet, Köseoğlu Cemal, Tunçer Eşref, Demir Erdem, Yüksel Gülhan, Altın Cihan, Kanadaşı Mehmet, Aksakal Aytekin, Oğuz Aytekin, Arkan Tuğba, Bilgi Mustafa, Kamberoğlu Seyfi, Gülsün Mehmet Sıdık, Çakır Murat, Şahin Nedim, Sakar Mehmet, Akyer Erdal, Karaca Elyesa, Eralp Ahmet Bülent, Bozkuş Rıfat, Develioğlu Hülya, Ocak Serin Sibel, Özer Necla, Şeker Taner, Koç Mevlüt, Sağ Saim, Çabuk Ali Kemal, Üstün Emel, Şirin Yusuf, Uçar Ender, Ergene Asım Oktay, Dursun Hüseyin, Alioğlu Emin, Kilci Hakan, Ertem Ahmet Göktuğ, Aytekin Saide, Yeter Ekrem, Şahin Alparslan, Bayram Fahri, Özentürk Cihan, Dal Kürşat, Çiçek Sümeyye, Doğan Cemile Elvin, Yiğit Sevil, Mazı Emrah Erkan, Kaya Emine, Şengün Ahmet, Okur Hasan Caner, Terzioğlu Nevin, Aktürk Müjde Yeşim, Şahin İbrahim, Acar Bilal, Atay Ahmet Engin, Yılmaz Ozan, İhsan Habiboğlu, Demirtunç Refik, Çetin Nezih Abdullah, Bambul Nail, Cengiz Mahir, Karakaş Mustafa Serkan, Özdemir Zeynep Tuğba, Kayıkçıoğlu Sezgi.

**Ukraine:** Olga Garkavenko, Lilia Kisilevych, Vadym Romanov, Olena Matova, Mariia Egorova, Inna Kovaleva, Evgen Andreev, Oleksandr Galetsky, Iryna Chulaevska, Iryna Sichkaruk, Olena Chirva, Pavlo Kaplan, Oleg Leshchuk, Olga Verbovska, Olena Karlinska, Ivanna Antoniuk-Scheglova, Hanna Havalko, Viktoria Potaskalova, Olena Karmazina, Oksana Onoprienko, Tetiana Ostashevska, Liudmyla Petrenko, Viktoria Bugeruk, Iryna Zaplatynska, Liudmyla Mazurok.

Table S1 **Variables tested but not significant at the 5% level in the multivariable logistic regression analysis, and therefore, not retained in the final model**

| **Variable** | ***p*-Value** |
| --- | --- |
| Body mass index (classes) | 0.9300 |
| Chronic inflammatory diseases | 0.8764 |
| Physical activity | 0.8102 |
| Metabolic syndrome defined according to ATP III | 0.7726 |
| Familial hypercholesterolemia diagnosis (Y/N) | 0.7811 |
| HDL-C at time of first diagnosis - categorized | 0.6818 |
| Diabetes type II | 0.6689 |
| Gender | 0.6359 |
| Hypertension | 0.4813 |
| Diagnosis or history of dyslipidemia | 0.4514 |
| Metabolic syndrome defined according to the IDF | 0.4587 |
| Waist circumference (IDF criteria) | 0.7238 |
| LDL-C target level | 0.7888 |
| Practice / Office | 0.9923 |
| History of chronic kidney disease | 0.4467 |
| Cancer | 0.3769 |
| Chronic obstructive pulmonary disease | 0.2052 |
| The investigator follows specific guidelines or recommendations for the management of lipid disorders | 0.1813 |
| Gender | 0.1179 |
| Diabetes | 0.1201 |
| Age group | 0.0920 |
| Years of practice | 0.0969 |
| Percentage of patients with dyslipidemia and/or with lipid-modifying treatments seen per day > 50.0 (median) | 0.0732 |
| Type of health insurance | 0.2726 |
| LDL-C at time of first diagnosis - categorized | 0.2007 |
| Private clinic | 0.1437 |

ATP, adult treatment panel; HDL-C, high-density lipoprotein cholesterol; IDF, International Diabetes Federation; LDL-C, low-density lipoprotein cholesterol

Table S2 Duration of hospitalizations in the 12 months before enrolment by risk level and LDL-C goal achievement (imputing 0 if no hospitalization)

|  | **Risk Level** | | | | | **LDL-C Goal Achieved** | | **All (*N* = 9049)** |
| --- | --- | --- | --- | --- | --- | --- | --- | --- |
|  | **Low Risk *(n* = 70)** | **Moderate Risk (*n* = 411** | **High Risk (*n* = 2621)** | **Very-High risk (*n* = 4842)** | **Risk Non-Assessable (*n* = 1105)** | **Yes (*n* = 3140)** | **No (*n* = 4734)** |  |
| Durations (days) of last three hospitalizations^a^ | *n* = 65 | *n* = 399 | *n* = 2457 | *n* = 4665 | *n* = 1031 | *n* = 2976 | *n* = 4545 | n= 8617 |
| Mean ± SD | 0.2±0.9 | 0.6±2.5 | 0.5±2.4 | 2.6±7.8 | 0.7±2.9 | 1.6±8.3 | 1.9±4.8 | 1.6 ±6.1 |
| Median (IQR) | 0.0 (0.0–0.0) | 0.0 (0.0–0.0) | 0.0 (0.0–0.0) | 0.0 (0.0–3.0) | 0.0 (0.0–0.0) | 0.0 (0.0–0.0) | 0.0 (0.0–0.0) | 0.0 (0.0–0.0) |
| Durations (days) of last three hospitalizations^a^ |  |  |  |  |  |  |  |  |
| For myocardial infarction | *n* = 65 | *n* = 400 | *n* = 2459 | *n* = 4698 | *n* = 1032 | *n* = 2987 | *n* = 4570 | *n* = 8654 |
| Mean ± SD | 0.0±0.0 | 0.0±0.0 | 0.0±0.5 | 0.4±2.2 | 0.0±0.0 | 0.3±1.8 | 0.3±1.7 | 0.2±1.6 |
| Median (IQR) | 0.0 (0.0–0.0) | 0.0 (0.0–0.0) | 0.0 (0.0–0.0) | 0.0 (0.0–0.0) | 0.0 (0.0–0.0) | 0.0 (0.0–0.0) | 0.0 (0.0–0.0) | 0.0 (0.0–0.0) |
| For unstable angina | *n* = 65 | *n* = 400 | *n* =2459 | *n* = 4708 | *n* = 1033 | *n* = 2991 | *n* = 4576 | *n* = 8665 |
| Mean ± SD | 0.0±0.0 | 0.0±0.2 | 0.0±0.5 | 0.4±1.8 | 0.0±0.5 | 0.2±1.3 | 0.3±1.6 | 0.2±1.4 |
| Median (IQR) | 0.0 (0.0–0.0) | 0.0 (0.0–0.0) | 0.0 (0.0–0.0) | 0.0 (0.0–0.0) | 0.0 (0.0–0.0) | 0.0 (0.0–0.0) | 0.0 (0.0–0.0) | 0.0 (0.0–0.0) |
| For ischemic stroke | *n* = 65 | *n* = 400 | *n* = 2459 | *n* = 4709 | *n* = 1033 | *n* = 2991 | *n* = 4577 | *n* = 8666 |
| Mean ± SD | 0.0±0.0 | 0.0±0.0 | 0.0±0.0 | 0.2±2.1 | 0.0±0.5 | 0.1±2.0 | 0.1±1.4 | 0.1±1.6 |
| Median (IQR) | 0.0 (0.0–0.0) | 0.0 (0.0–0.0) | 0.0 (0.0–0.0) | 0.0 (0.0–0.0) | 0.0 (0.0–0.0) | 0.0 (0.0–0.0) | 0.0 (0.0–0.0) | 0.0 (0.0–0.0) |
| For coronary revascularization | *n* = 65 | *n* = 400 | *n* =2459 | *n* = 4707 | *n* = 1033 | *n* = 2991 | *n* = 4575 | *n* = 8664 |
| Mean ± SD | 0.0±0.0 | 0.0±0.0 | 0.0±0.1 | 0.2±1.6 | 0.0±0.0 | 0.1±1.3 | 0.2±1.2 | 0.1±1.2 |
| Median (IQR) | 0.0 (0.0–0.0) | 0.0 (0.0–0.0) | 0.0 (0.0–0.0) | 0.0 (0.0–0.0) | 0.0 (0.0–0.0) | 0.0 (0.0–0.0) | 0.0 (0.0–0.0) | 0.0 (0.0–0.0) |

^a^Mean of last three hospitalizations
IQR, interquartile range; LDL-C, low-density lipoprotein cholesterol; n, number of patients in the sample population; SD, standard deviation
